# Comparison of tobacco import and tobacco control in five countries: lessons learned for Indonesia

**DOI:** 10.1186/s12992-020-00595-y

**Published:** 2020-07-18

**Authors:** Abdillah Ahsan, Nur Hadi Wiyono, Meita Veruswati, Nadhila Adani, Dian Kusuma, Nadira Amalia

**Affiliations:** 1 Faculty of Economics and Business, University of Indonesia, Jl. Margonda Raya, Pondok Cina, Kecamatan Beji, Kota Depok, Jawa Barat 16424 Indonesia; 2 Department of Public Health Sciences, University of Muhammadiyah Prof. Dr. HAMKA, Jakarta, Indonesia; 3 Centre for Health Economics and Policy Innovation, Imperial College Business School, London, UK; 4 Faculty of Economics and Administration, University of Malaya, Kuala Lumpur, Malaysia

**Keywords:** Tobacco import, Tobacco control, Indonesia

## Abstract

**Background:**

With a 264 million population and the second highest male smoking prevalence in the world, Indonesia hosted over 60 million smokers in 2018. However, the government still has not ratified the Framework Convention on Tobacco Control. In the meantime, tobacco import increases rapidly in Indonesia. These create a double, public health and economic burden for Indonesia’s welfare.

**Objective:**

Our study analyzed the trend of tobacco import in five countries: Indonesia, Pakistan, Bangladesh, Zimbabwe, and Mozambique. Also, we analyze the tobacco control policies implemented in these countries and determine some lessons learn for Indonesia.

**Methods:**

We conducted quantitative analyses on tobacco production, consumption, export, and import during 1990–2016 in the five countries. Data were analyzed using simple ordinary least square regressions, correcting for time series autocorrelation. We also conducted a desk review on the tobacco control policies implemented in the five countries.

**Results:**

While local production decreased by almost 20% during 1990–2016, the proportion of tobacco imports out of domestic production quadrupled from 17 to 65%. Similarly, the ratio of tobacco imports to exports reversed from 0.7 (i.e., exports were higher) to 2.9 (i.e., import were 2.9 times higher than export) in 1990 and 2016, respectively. This condition is quite different from the other four respective countries in the observation where their tobacco export is higher than the import. From the tobacco control point of view, the four other countries have ratified the Framework Convention on Tobacco Control (FCTC).

**Conclusion:**

The situation is unlikely for Indonesia to either reduce tobacco consumption or improve the local tobacco farmer’s welfare, considering that the number of imports continued to increase. Emulating from the four countries, Indonesia must ratify the FCTC and implement stricter tobacco control policies to decrease tobacco consumption and import.

## Background

With a 264 million population and the second highest male smoking prevalence in the world, Indonesia hosted over 60 million smokers in 2018 [1]. This number is increasing as the tobacco consumption prevalence (smoking and chewing) among aged 15+ years remained high at 34% in 2018. The male tobacco consumption prevalence was very high, at 63% in 2018. Among the youth 10–18 years, the smoking prevalence is 9.1% increased by almost 30% during 2013–2018 [2]. All this contributes to the high burden of cardiovascular diseases. The Indonesian Global Burden of Disease study showed ischemic heart disease and stroke as the top two leading causes of death and disability in 2016 [3]. Despite all this, the national tobacco control programs are very limited in Indonesia, partly due to not yet ratifying the Framework Convention on Tobacco Control (FCTC) [4–6].

One main argument against comprehensive tobacco control in Indonesia is the welfare and livelihood of tobacco farmers. It is believed that any tobacco control efforts would decrease the amount of tobacco consumed, and local tobacco farmers would be negatively affected. Data shows that the tobacco industry has experienced steady growth over time. Domestic cigarette manufacturing increased by 54% from 222 billion sticks in 2005 to 342 billion sticks in 2016. However, it has been a different story for local farmers. Local tobacco production decreased by 17% from 153,000 tons in 2005 to 127,000 tons in 2016 [7]. These gaps between production and consumption have been filled by imported, usually cheaper, tobacco, which contributes to reducing the welfare of tobacco farmers. Increasing tobacco imports, together with other imports, also hurt economic growth.

Hence, Indonesia experiences the double burden of welfare from increasing tobacco consumption that decreases the public health, and rising tobacco import that reduces the economic growth and farmers’ well-being. As a developing country, Indonesia can learn from other developing countries who has a better situation, lower tobacco consumption, and lower tobacco import. This study aims to analyze the trend of tobacco import, and tobacco consumption of Indonesia compare to four other developing countries and determine what Indonesia can learn from them.

## Methods

We conducted trend analyses in terms of volume (in tons) and value (in US$) of local production, local consumption, import, and export of tobacco during 1990–2016 in Indonesia, as 2016 was the latest available data from the Ministry of Agriculture Report in 2018, and the four other countries. Local production was defined as all tobacco produced domestically and used for consumption and exports. Local consumption was defined as all domestic tobacco use, including from local production and imports, and excluding exports. Data on Indonesia were obtained from the Indonesian Ministry of Agriculture, while data on the comparable countries were obtained from FAOSTAT [8]. Other countries were chosen for similarities in tobacco production and income level. Zimbabwe, Pakistan, and Bangladesh are lower-middle income countries, and Mozambique is a low-income country, as per World Bank definition.

We employed ordinary least square regressions, correcting for time series auto-correlations using Stata 15.1. Also, we conducted desk reviews on the implementation of tobacco control regulations in the four countries compare to Indonesia’s situation.

## Results

### Local production, export, and import of tobacco

**Fig. 1:**
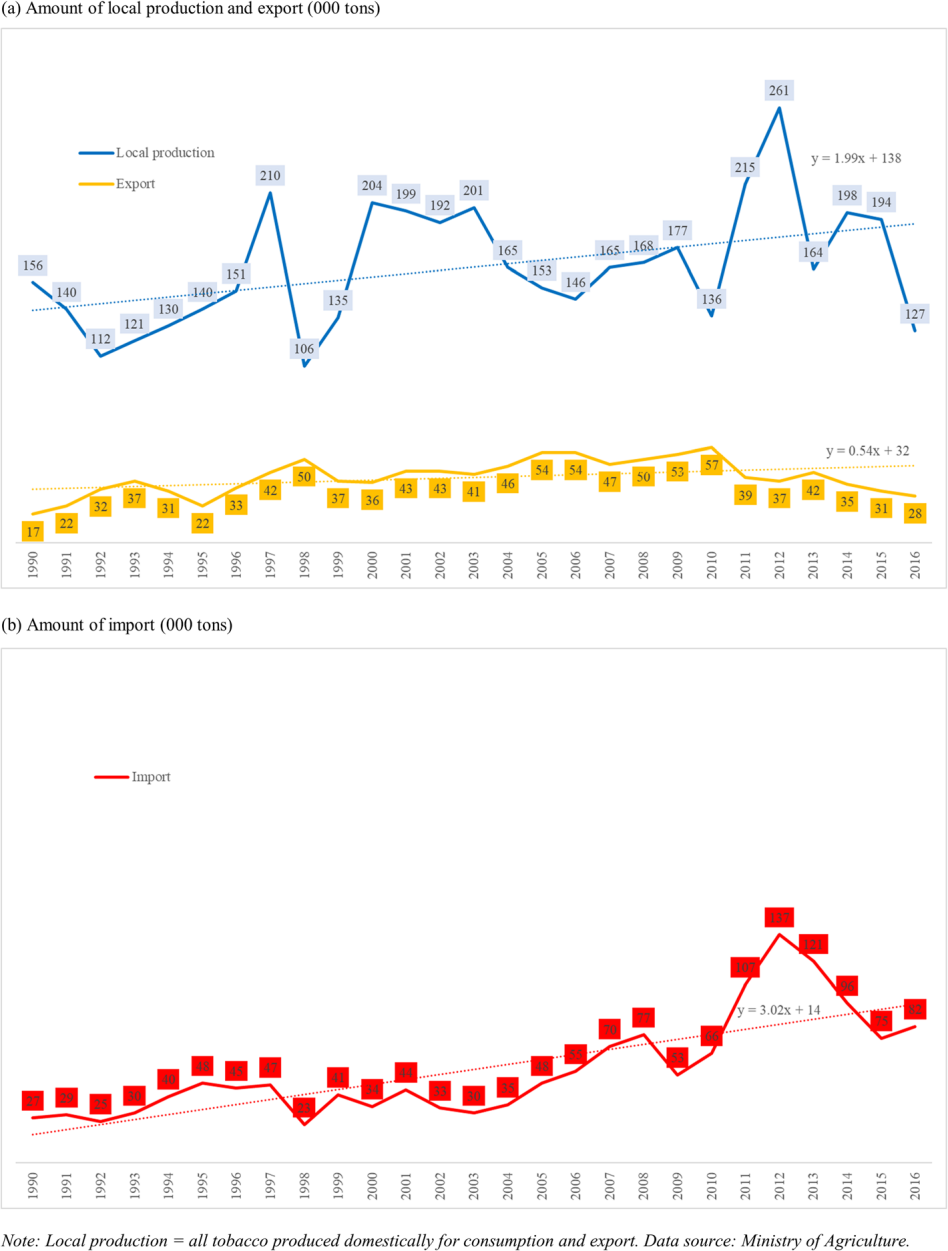
Local production of tobacco, export, and import in Indonesia 1990–2016

Figure 1 shows the level of and trends in the local production of tobacco and export (panel a) and import (panel b) during 1990–2016. While indicating an increasing trend during the period, the local production level decreased from 156,000 tons in 1990 to 127,000 tons in 2016. While the level of export increased from 17,000 tons in 1990 to 28,000 tons in 2016, the increasing trend had a lower slope. The level of export peaked at 57,000 tons in 2010, and the trend has been decreasing since then. In the meantime, the level of import increased from 27,000 tons in 1990 to 82,000 tons in 2016. The slopes of the import reveals an increasing trend (3.02) much steeper than that of export (0.54). The level of imports peaked at 137,000 tons in 2012, with a rising trend from 2010 to 2016.

**Fig. 2:**
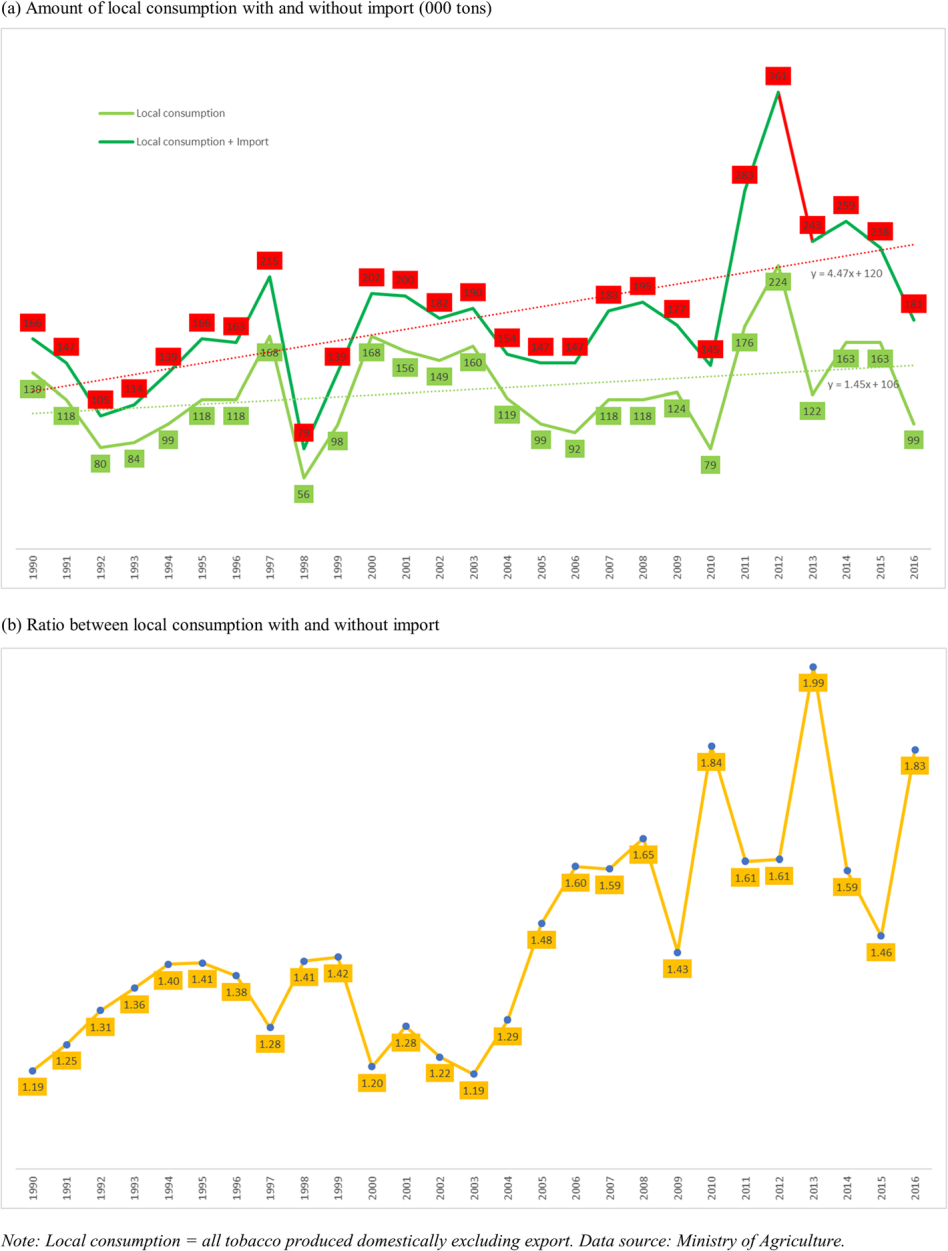
Local consumption of tobacco, import, and ratio in Indonesia 1990–2016

Figure 2 shows the local tobacco consumption with and without import in terms of level (panel a) and ratio (panel b) during 1990–2016. In this figure, we removed export from local production. Without import, while showing an increasing trend during the period, local consumption level decreased from 139,000 tons in 1990 to 99,000 tons in 2016. The scale was lowest at 56,000 tons in 1998 and highest at 224,000 tons in 2012. With imports, the local consumption level increased from 166,000 tons in 1990 to 181,000 tons in 2016. the slope of this is increasing trend (4.47) and much steeper than that of without import (1.45). The level was lowest at 79,000 tons in 1998 and highest at 361,000 tons in 2012. Furthermore, the ratio between local consumption with and without import increased from 1.19 in 1990 to 1.83 in 2016. In other words, import added 19 and 83% to domestic consumption in 1990 and 2016, respectively. The ratio peaked at 1.99 in 2013, showing that import added 99% to local use (almost double).

**Fig. 3:**
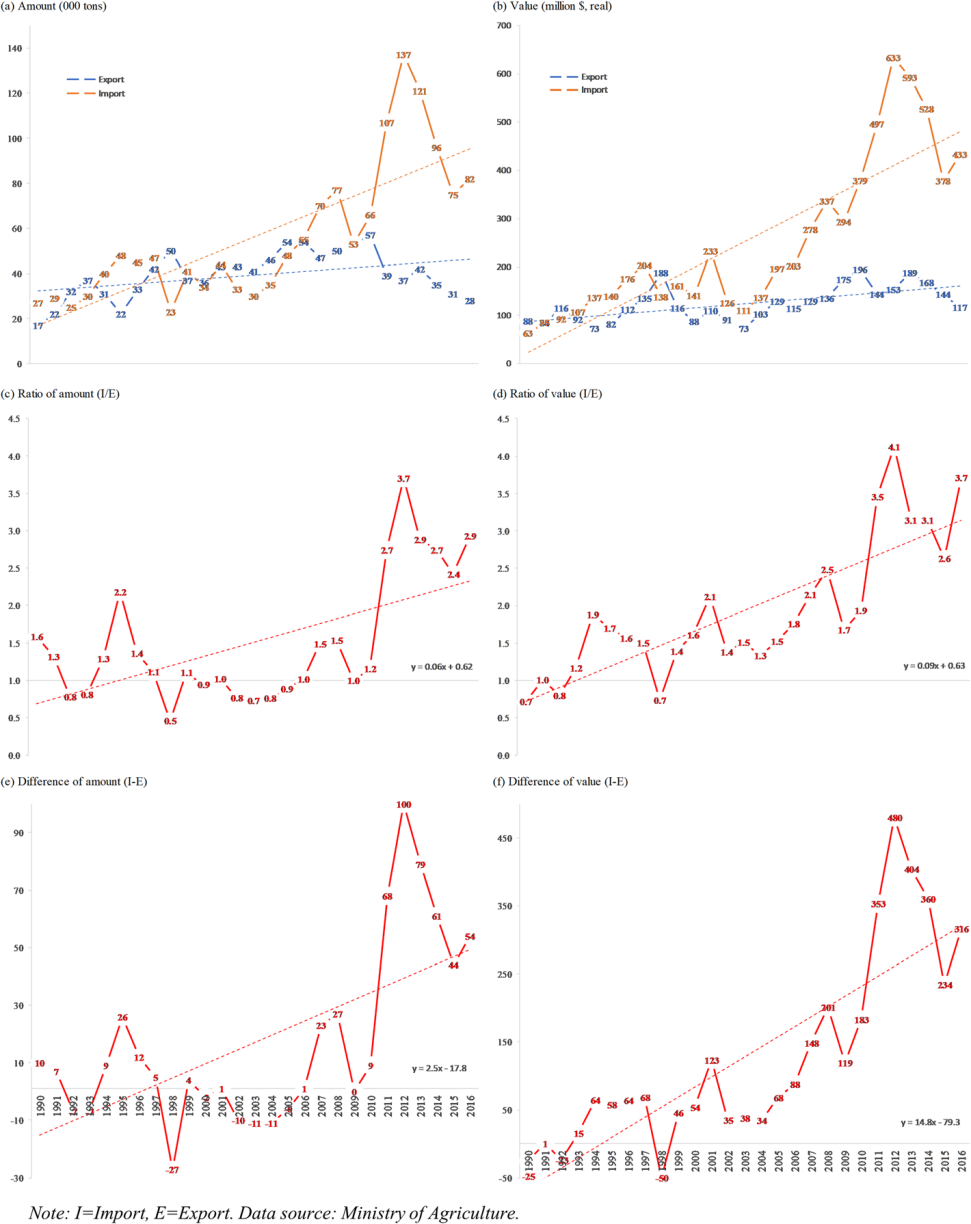
Volume (000 tons) and value (million US$) of import and export of tobacco in Indonesia 1990–2016

Figure 3 shows a direct comparison between export and import in terms of the amount (000 tons) and monetary value (million $, real) during 1990–2016. The amount (volume) of imports has been significantly increasing, but that of exports has not (panel a). Number of imports tripled from 27,000 to 82,000 tons. Besides, exports increased only slightly from 17,000 to 28,000 in 1990 and 2016, respectively. Notably, the number of imports peaked at 137,000 tons in 2012 (a five-time increase from that in 1990). The drop after 2013 probably due to the dramatic decline in consumption after the release of Government Regulation 109/2012 concerning Control of Materials that Contain Addictive Substances in Tobacco Products in the Interests of Health. The trends were similar in terms of the value of imports and exports (panel b). The ratios between imports and exports have also been significantly increasing (panels c-d). The ratios of import volume were 1.6 and 2.9 times higher than that of export volume in 1990 and 2016, respectively. The trends were similar for the ratio between import and export value. The ratios of import value were 0.7 (i.e., export value was 1.4 times higher) and 3.7 times higher than that of export value during 1990–2016. The differences between import and export have also been significantly increasing (panels e-f). The differences between import and export volume were 10,000 and 54,000 tons in 1990 and 2016, respectively. The trends were similar to the differences between import and export values. The differences between import and export value were -$25 million (i.e., import value was 25 million higher) and 316 million in 1990 and 2016, respectively.

To further understand the increasing imports, Table 1 shows the volume and value of imported tobacco in 2016. In terms of amount, out of 82,000 tons of imported tobacco, 42,000 tons (51%) were Virginia tobacco, 13,000 tons (16%) were Oriental tobacco, 14,000 tons (17%) were other tobacco, 6000 tons (7%) were Burley tobacco, and 7000 tons (9%) were tobacco stems and refuse. Among all the tobacco leaf, Virginia tobacco is the highest demanded tobacco leaf. Nevertheless, the local production still cannot meet the demand. As an implication, the number of tobaccos import for this kind of tobacco will remain high as it will be difficult to substitute it. In monetary value, the proportions are similar except for tobacco stems and refuse, which was only 1% of total imported values. In more detail, the primary type of Virginia, Oriental, and Burley tobacco that were imported was “partly or wholly stemmed/stripped”.

**Table 1:** Volume and value of imported tobacco 2016

	Volume		Value	
	000 Tons	% of TOTAL	$m	% of TOTAL
Virginia tobacco	42	51%	240	50%
Oriental tobacco	13	16%	95	20%
Other tobacco	14	17%	100	21%
Burley tobacco	6	7%	36	8%
Tobacco stems & refuse	7	9%	6	1%
TOTAL	82		477	
Virginia tobacco				
Partly or wholly stemmed/stripped, flue-cured	39	48%	216	45%
Partly or wholly stemmed/stripped, not flue-cured	1	1%	4	1%
Not stemmed/stripped, flue-cured	2	2%	21	4%
Not stemmed/stripped, not flue-cured	0.00	0%	0.00	0%
Oriental tobacco				
Partly or wholly stemmed/stripped	13	16%	95	20%
Burley tobacco				
Partly or wholly stemmed /stripped	6	7%	36	8%
Not stemmed/stripped	0.05	0%	0.19	0%
Other tobacco				
Not stemmed/stripped, not flue-cured	7	9%	49	10%
Not stemmed/stripped, flue-cured	1	1%	3	1%
Partly or wholly stemmed stripped, not flue-cured	4	5%	36	8%
Partly or wholly stemmed stripped, flue-cured	2	2%	12	2%
Tobacco stems & refuse				
Stems	6	7%	5	1%
Refuse	1	2%	1	0%
TOTAL	82		477	

Volume in thousand tons; Value nominal 2016 in million US$. Data source: Ministry of Agriculture

Table 2 compares the production, export, and import between Indonesia and other countries in 2016. These countries were chosen, given similarities in terms of local production and country income level. In this list, compared to Indonesia, Zimbabwe had more production with 169,000 tons; Zimbabwe and Mozambique had more exports with 155,000 and 83,000 tons, respectively; no country had more imports. Indonesia had the highest ratio of import to export at 293% (i.e. the amount of import was almost three times that of export). That ratio in comparator countries ranged from 1% in Mozambique and Bangladesh to 67% in Pakistan.

**Table 2:** Tobacco production, export, and import in Indonesia and comparator countries 2016

Country	Local produc-tion	Export	Import	Ratio Import/Export	Local consump-tion	Local cons + import	Ratio local cons. Without / with import
	[1]	[2]	[3]	[4] = [3/2]	[5] = [1, 2]	[6] = [5 + 3]	[7] = [6/5]
Indonesia	127	28	82	2.93	99	181	1.83
Zimbabwe	169	155	3	0.02	14	17	1.21
Pakistan	116	3	2	0.67	113	115	1.02
Mozambique	93	83	1	0.01	10	11	1.10
Bangladesh	88	20	1	0.05	68	69	1.01

Local production, export, and import are in thousand tons. Cons = consumption. Comparable countries have similarities in local production and income level (all lower-middle income countries, except for Mozambique a low-income country, as per World Bank definition). Data source: FAOSTAT

Moreover, Pakistan had the highest local consumption without import (113,000 tons), but Indonesia had the highest domestic consumption after adding import (181,000 tons). The ratio of local consumption without and with adding the import was highest in Indonesia at 1.83. That ratio in comparator countries ranged from 1.01 in Bangladesh to 2.21 in Zimbabwe.

## Discussion

While one main argument against tobacco control in Indonesia is the welfare of farmers from local consumption and export, our findings show that is not the case. During 1990–2016, domestic production and export were relatively stagnant while import increased rapidly. More recently, export has been much lower than import mainly when the ratio between import and export was 2.93 (i.e. the import amount was 293% of export) in 2016. In effect, local cigarette manufacturing relied more and more on import with 83% of all domestic consumption were from import.

The situation is quite different in other major tobacco-producing countries. In Zimbabwe, Mozambique, and Bangladesh, the ratio of import and export ranged from 0.01 to 0.05. In other words, the import amount was only 1–5% of export in those countries – compared to 293% in Indonesia. Also, local tobacco consumption did not rely so much on import. In Bangladesh, Pakistan, and Mozambique, the ratio of domestic consumption without and with adding imports ranged from 1.01 to 1.10. In other words, import contributed only 1–10% of total tobacco consumption in those countries – compared to 83% in Indonesia [8, 9].

Zimbabwe is among the highest exporters of tobacco raw materials globally. Zimbabwe contributed up to 4.1% of total global raw tobacco export in 2018 [10]. Tobacco is the 2nd largest export commodity of the country, sharing 19.1% of the country’s total export [11]. Nevertheless, the smoking prevalence in Zimbabwe is only 14.1% in 2018 [12]. This fact has shown that amid being one of the largest tobacco producers, Zimbabwe can prevent their citizen from smoking.

Moreover, despite the low number of consumers, Zimbabwe still implements the important policy in tobacco control, increase the price of cigarettes and cigarette has become less affordable since 2008 [12]. Even though the other FCTC policies in Zimbabwe remain low and moderate, but the two policies will remain relevant as disincentives for both local and import consumption. The same applied to Mozambique, where the smoking prevalence is slightly higher – 14.9% [13]. As a net tobacco exporter country where tobacco accounted for 4.15% of the country’s export – the 4th largest export commodity [14], the cigarette price in Mozambique also increased by 85% in nominal terms in 2013–2016 [15], making the cigarette less affordable.

The other essential note from the countries being observed is that Bangladesh, amid being the country with the highest smoking prevalence (39.5%), has the lowest number of local tobacco production. It is important to note that among the other four countries that have ratified the FCTC, Bangladesh is the only country that has supported the viable alternative to tobacco agriculture and hence might decrease the local tobacco production [16]. Nevertheless, the domestic tobacco demand shown by the local tobacco consumption in Bangladesh is only 1/3 of the local use in Indonesia. This fact indicates that even though the number of local production is low in Bangladesh, there will be no need to increase import since there is no demand from the consumption side.

Moreover, the implementation of FCTC Protocol in Bangladesh is quite exemplary, compared to the other countries observed. Bangladesh, among the observed countries, is the only country to impose the tobacco excise tax above the minimum WHO benchmark of 70%. Bangladesh has imposed tobacco excise tax as much as 71% – highest among the other four countries [17]. This policy has made the cigarette less affordable in the country and has probably pressed the volume of tobacco consumption in the country. Also, Pakistan, with 20.7% of smoking prevalence in 2018, is relatively strong in terms of non-monetary FCTC Protocol implementation compared to the other four countries [18]. Based on the WHO Report, Pakistan has fully implemented the policies on smoke-free environments as well as health warnings and anti-tobacco campaigns. All these implementations in other countries could be valuable lessons learned for Indonesia.

The unfavorable condition in Indonesia is primarily due to the lack of trade barriers, both non-tariff and tariff. Indonesia is a member of the ASEAN Free Trade Area (AFTA) since 1990 and the ASEAN-China Free Trade Area (ACFTA) since 2015). As a member, Indonesia agreed to reduce tariff barriers on almost all products and services, including tobacco and tobacco products. Within AFTA, import tariffs of raw tobacco from member countries are reduced to 5% and to be further reduced to 0%. Within ACFTA, import tariff was 10% during 2015–2017 and reduced to 5% in 2018. Relative to other countries in the region, tobacco import tariffs in Indonesia are relatively low at 4.4%, compared to 15% in Thailand, 29% in Vietnam, 40% in Laos, and 178% in Malaysia [19]. This situation makes Indonesia an attractive destination for other countries to export.

Indonesia lacks comprehensive tobacco control efforts as opposed to the other four countries in the previous discussion. Increasing tobacco consumption is a favorable condition for tobacco import in Indonesia. The clear winner is the tobacco industry, which has experienced a dramatic increase in cigarette production and cheaper tobacco imports.

## Conclusion

Indonesia, as the home for more than 61.4 Million tobacco users, has been a huge potential market for the tobacco industry to sell their products. The high consumption of tobacco products has forced the tobacco industry to meet the demand both by producing locally or through imports. During the past decades, tobacco imports in Indonesia have dramatically increased, reaching up to 2.93 times the number of tobacco export. This primarily due to the inability of local productions to meet the demand. This condition is unfavorable for both local farmers and public health, especially the tobacco control situation. However, this should be unnecessary if Indonesia can significantly reduce the number of tobacco use and, hence, the tobacco import. Emulated to the four countries being analyzed, Indonesia must increase its tobacco control policies and implementation. Some of the notable strategies documented in the studies are increasing tobacco tax significantly and improving the non-monetary tobacco control policies like enforcing smoke-free area regulation and ample pictorial health warning. Nevertheless, this would be difficult to achieve if Indonesia is still not ratifying the FCTC since it will be hard to enforce the implementation.

## Data Availability

Data is available upon request to: Dian Kusuma (d.kusuma@imperial.ac.uk).
